# Optical coherence tomography-guided versus angiography-guided percutaneous coronary intervention in acute coronary syndrome: a meta-analysis

**DOI:** 10.1007/s00392-023-02272-7

**Published:** 2023-07-31

**Authors:** S. Macherey-Meyer, M. M. Meertens, S. Heyne, S. Braumann, T. Tichelbäcker, H. Wienemann, V. Mauri, S. Baldus, C. Adler, S. Lee

**Affiliations:** Faculty of Medicine, Clinic III for Internal Medicine, University of Cologne, University Hospital Cologne, Kerpener Straße 62, 50937 Cologne, Germany

**Keywords:** Optical coherence tomography, OCT, STEMI, NSTE-ACS, Percutaneous coronary intervention, PCI, Angiography

## Abstract

**Background:**

Percutaneous coronary intervention (PCI) is standard of care in patients with acute coronary syndrome (ACS) suitable for interventional revascularization. Intracoronary imaging by optical coherence tomography (OCT) expanded treatment approaches adding diagnostic information and contributing to stent optimization.

**Objectives:**

This meta-analysis aimed to assess the effects of OCT-guided vs. angiography-guided PCI in treatment of ACS.

**Methods:**

A structured literature search was performed. All controlled trials evaluating OCT-guided vs. angiography-guided PCI in patients with ACS were eligible. The primary end point was major adverse cardiac events (MACE).

**Results:**

Eight studies enrolling 2612 patients with ACS were eligible. 1263 patients underwent OCT-guided and 1,349 patients angiography-guided PCI. OCT guidance was associated with a 30% lower likelihood of MACE (OR 0.70, 95% CI 0.53–0.93, *p* = 0.01, *I*^2^ = 1%). OCT-guided PCI was also associated with significantly decreased cardiac mortality (OR 0.49, 95% CI 0.25–0.96, *p* = 0.04, *I*^2^ = 0%). There was no detectable difference in all-cause mortality (OR 1.08, 95% CI 0.51–2.31, *p* = 0.83, *I*^2^ = 0). Patients in OCT-guided group less frequently required target lesion revascularization (OR 0.26, 95% CI 0.07–0.95, *p* = 0.04, *I*^2^ = 0%). Analysis of myocardial infarction did not result in significant treatment differences. In subgroup or sensitivity analysis the observed advantages of OCT-guided PCI were not replicable.

**Conclusion:**

The evidence suggests that PCI guidance with OCT in ACS decreases MACE, cardiac death and target lesion revascularization compared to angiography. On individual study level, in subgroup or sensitivity analyses these advantages were not thoroughly replicable.

**Supplementary Information:**

The online version contains supplementary material available at 10.1007/s00392-023-02272-7.

## Introduction

Acute coronary syndrome (ACS) is a life-threatening disease with high morbidity and mortality burden. Immediate patient management is required, and invasive coronary angiography remains the diagnostic standard [1, 2]. Coronary angiography allows adequate treatment by percutaneous coronary intervention (PCI) [1, 2]. Balloon angioplasty followed by stent implantation is the predominant revascularization strategy, but in selected cases balloon angioplasty only, thrombus aspiration or conservative management is preferred over stent deployment [1, 3–5]. For decades, stent implantation was based only on fluoroscopic findings [6]. Quantitative standards and measurements were limited until recent technical developments in intracoronary imaging or functional assessment expanded the interventional repertoire [6]. Imaging-guided PCI with stent implantation in an elective setting was advantageous compared to conventional angiography resulting in a significant reduction of cardiac death and major adverse cardiac events previously [7]. Optical coherence tomography (OCT) and intravascular ultrasonography (IVUS) are the most frequently used imaging techniques [6, 8].

OCT is a light-based imaging technique in near-infrared spectrum with excellent near-field imaging, but technically axial range is limited and use depends on blushing the examined artery [9–11]. OCT allows detection of the underlying pathology of ACS, for example, plaque rupture can be distinguished from plaque erosion [10]. Precise measurements and visualization enable defining landing zone or optimal sizing in infarct-related arteries. After stent deployment, OCT may diagnose edge dissection, stent underexpansion, malapposition, or residual disease [10]. The applicability of OCT in PCI was demonstrated in elective circumstances before, but data on efficacy under urgent conditions during ACS are limited [7, 12, 13]. The current meta-analysis aimed to comprehensively assess the effects of OCT-guided compared to angiography-guided PCI in ACS based on efficacy outcomes.

## Methods

This meta-analysis was conducted using a pre-specified protocol and reproducible plan for literature search and synthesis according to the Preferred Reporting Items for Systematic reviews and Meta-Analysis (PRISMA) guidelines [14].

Controlled trials comparing OCT-guided to angiography-guided PCI in patients with ACS requiring interventional revascularization were included. Randomized controlled trials (RCT) and non-randomized controlled studies (NRS) were eligible. All details regarding search strategy, data extraction, and study selection are in presented in suppl. material 1.

The primary end point was major adverse cardiac events (MACE), a composite of cardiac mortality, myocardial infarction, and target vessel revascularization (TVR). The individual components of MACE, all-cause mortality, and target lesion revascularization (TLR) were secondary efficacy endpoints.

Risk of bias at study level was assessed using the Cochrane Collaborations risk of bias tool (RoB2, version 08/22/2019) for randomized trials [15]. Non-randomized controlled studies were assessed using the Cochrane Collaborations Risk Of Bias In Non-randomized Studies of Interventions (ROBINS-I, version 10/20/2016) tool [16]. Risk of bias assessment was performed by two individual investigators (SMM, SH). In case of discrepancy a third independent investigator was consulted (MMM).

Random-effects meta-analyses were performed using the Mantel–Haenszel method for dichotomous data. Pooled odds ratios (ORs) and 95% confidence intervals (CI) are given for each analysis with a two-sided significance level of *p* < 0.05 (RevMan 5.3, Nordic Cochrane Centre, Cochrane Collaboration). The extent of heterogeneity was approximated by *I*^2^ tests considering 0–40% as non-important, 30–60% as moderate, 50–90% as substantial, and 75–100% as considerable heterogeneity. Pre-specified analysis of publication bias by funnel plot was not appropriately feasible given the low number of studies included.

Follow-up varied between the trials and timing of measurement of outcomes during the study period was heterogeneously performed. Consequently, ORs were calculated from event data of longest follow-up of each study. Post hoc subgroup analyses were performed to test whether timing of OCT (OCT pre- or post-stent implantation) affected the results. This approach allowed differentiation between the use of OCT as diagnostic tool compared to stent optimization and subsequent therapeutically consequences.

Post hoc sensitivity meta-analyses were performed according to risk of bias judgement to assess the impact of study quality on the investigated outcomes. RCTs with “high” risk of overall bias were excluded from sensitivity analysis. NRS with “serious” or “critical” risk of overall bias were eliminated from sensitivity analyses.

We did not obtain ethical approval for this meta-analysis because we did not collect data from individual human subjects.

## Results

### Study selection

**Fig. 1:**
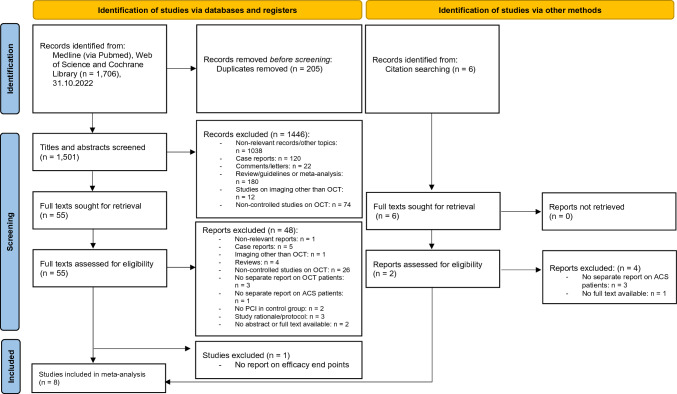
PRISMA flow chart diagram. *OCT* optical coherence tomography, *PCI* percutaneous coronary intervention, *ACS* acute coronary syndrome

A total of 1706 articles were identified by the described search strategy (see Fig. 1, PRISMA Flow chart). After removing duplicates, titles and abstracts of 1501 remaining articles were screened. 1446 articles were excluded which left 55 references for assessment of the full-text articles. Six additional full texts were assessed for eligibility by handsearching. Considering exclusion criteria eight studies were finally included in quantitative analyses [17–24].

### Studies

Eight controlled studies were included in meta-analysis [17–24] (see Table 1). Of these, three were RCTs [18, 23, 24]. Four trials were conducted as case series studies with propensity score matching (PSM), notably D’Ascenzo et al. and Iannaccone et al. each conducted PSM pooling data from the OCT-FORMIDABLE registry [17, 19–21]. The lasting study was designed as case series study without matching [22]. The use of OCT varied in the trials: three studies used OCT preliminary to PCI as diagnostic tool [20–22], one trial investigated stent optimization and used OCT solely after PCI [17], and four studies used OCT both in the pre- and post-stent implantation period [18, 19, 23, 24].

**Table 1:** Characteristics of included studies and patients

		D’Ascenzo et al. [20]	Di Giorgio et al. [17]	Iannaccone et al. [21]	Khalifa et al. [22]	Meneveau et al. [18] DOCTORS	Jia et al. [23] EROSION III	Antonsen et al. [24] OCTACS	Sheth et al. [19] TOTAL
Study characteristics									
Study period		01/2014–10/2015 and 01/2009–12/2012	10/2009–02/2010	01/2014–10/2015 and 06/2009–10/2015	01/2009–12/2018	09/2013–12/2015	12/2017–11/2019	08/2011–05/2013	08/2010–07/2014
Study design		PSM analysis of registry data, multicentric study	PSM analysis, case series	PSM analysis of registry data, multicentric study	Case series	RCT	RCT	RCT	PSM analysis, case series
Comparator treatment		Angiography FFR guidance	Angiography	Angiography	Angiography	Angiography	Angiography	Angiography	Angiography
Follow-up period (median)		25 months	12 months	700 days	1 year	6 months	369 days	6 months	12 months
Baseline characteristics of patients included									
OCT group	Patients	197	40	270	260	120	112	50	214
Angiography group	Patients	197	40	270	130	120	114	50	428
OCT group	Age, median	63 (13)	67.9 (11.3)	60 (13)	70 (13)	60.8 (11.5)	54.5 (11.2)	61.8 (9.4)	60.9 (11.5)
Angiography group	Age, median	64 (10)	62.7 (10.9)	61 (12)	73 (11)	60.2 (11.3)	56.4 (10.4)	62.6 (11.0)	61.2 (12.1)
OCT group	Male patients	153 (77.7)	35 (87.5)	226 (79)	176 (68)	95 (79.2)	89 (79.5)	36 (72)	167 (78)
Angiography group	Male patients	151 (76.6)	31 (77.5)	227 (79)	90 (69)	91 (75.8)	91 (79.8)	34 (68)	354 (82.7)
OCT group	Hypertension	133 (67.5)	30 (75)	160 (56)	219 (84)	67 (55.8)	47 (42)	28 (56)	–
Angiography group	Hypertension	139 (70.6)	32 (80)	168 (59)	109 (84)	50 (41.7)	45 (39.5)	28 (56)	–
OCT group	Diabetes	38 (19.3)	12 (30)	49 (17)	101 (39)	26 (21.7)	29 (25.9)	8 (16)	38 (17.8)
Angiography group	Diabetes	48 (24.4)	13 (32.5)	48 (18)	61 (47)	19 (15.8)	19 (16.7)	5 (10)	79 (18.5)
OCT group	Smoking	113 (57.4)	18 (45)	162 (57)	78 (30)	47 (39.2)	64 (57.1)	23 (46)	93 (43.5)
Angiography group	Smoking	120 (60)	22 (55)	163 (58)	50 (38)	51 (42.5)	73 (64.0)	18 (36)	184 (43.0)
OCT group	Dyslipidemia	114 (57.9)	29 (72.5)	142 (49)	196 (75)	59 (49.2)	–	–	–
Angiography group	Dyslipidemia	109 (55.3)	19 (47.5)	135 (47)	97 (75)	56 (46.7)	–	–	–
Type of acute coronary syndrome and management									
OCT group	Unstable Angina	54 (27.4)	0	46 (16)	18 (7)	10 (8.3)	0	0	0
	NSTEMI	89 (45.2)	16 (40)	99 (34)	42 (16)	110 (91.7)	0	50 (100)	0
	STEMI	54 (27.4)	24 (60)	140 (49)	200 (77)	–	112 (100)	0	214 (100)
Stenting (% of patients)		Not separately reported	100	100	100	100	43.8	100	Not separately reported
Angiography group	Unstable Angina	53 (26.9)	0	42 (15)	15 (12)	9 (7.5)	0	0	0
	NSTEMI	80 (40.9)	21 (52.5)	93 (36)	18 (14)	111 (92.5)	0	50 (100)	0
	STEMI	65 (33)	19 (47.5)	150 (53)	97 (75)	–	114 (100)	0	428 (100)
Stenting (% of patients)		Not separately reported	100	100	100	100	58.5	100	Not separately reported

*OCT* optical coherence tomography, *PSM* propensity score matching, *RCT* randomized controlled trial; *FFR* fractional flow reserve, *NSTEMI* Non-ST segment elevation myocardial infarction, *STEMI* ST segment elevation myocardial infarction. (): standard deviation in reported ages, percentages in other rows

### Assessment of bias

Assessment of bias was performed in three RCTs using RoB2 tool [18, 23, 24]. Two RCTs were associated with “some concerns” in overall judgement (see Table 2). The OCTACS trial was judged to be at “high” risk of overall bias [24]. This judgement was mainly caused by substantial deviations from defined intervention because two patients underwent imaging using IVUS instead of OCT and one patient with peri-interventional complication likely caused by OCT was excluded from efficacy analysis.

**Table 2:** Risk of bias assessment of randomized controlled trials

Risk of bias	Randomization	Deviations from intended interventions	Missing outcome data	Measurement of the outcomes	Selection of the reported results	Overall risk of bias
DOCTORS [18]	Low	Some concerns	Low	Some concerns	Low	Some concerns
EROSION III [23]	Low	Some concerns	Low	Some concerns	Low	Some concerns
OCTACS [24]	Low	High	Some concerns	Some concerns	Some concerns	High

Potential sources of bias were assessed in five NRS by ROBINS-I tool [17, 19–22]. Three trials were judged to have moderate risk of overall bias, whereas two trials were considered to have critical risk of overall bias (see Table 3). The latter were included in the overall analysis, but were precluded from sensitivity analysis. The overall judgement “critical” risk of bias was based on severe differences in patient selection and varying treatment periods and protocols between OCT and comparator group [17, 22].

**Table 3:** Risk of bias assessment of non-randomized controlled studies

Risk of bias	Confounding	Patient selection	Classification of intervention	Deviations from the intended interventions	Missing data	Outcome measurement	Selection of the reported results	Overall risk of bias
D’Ascenzo et al. [20]	Moderate	Moderate	Low	Low	Low	Low	Moderate	Moderate
Di Giorgio et al. [17]	Critical	Critical	Low	Low	Moderate	Low	Moderate	Critical
Iannaccone et al. [21]	Moderate	Moderate	Low	Low	Low	Low	Moderate	Moderate
Khalifa et al. [22]	Serious	Critical	Low	Low	Low	Serious	Moderate	Critical
Sheth et al. [19]	Moderate	Moderate	Low	Low	Moderate	Low	Low	Moderate

### Patient-level baseline characteristics and procedural data

A total of 2612 patients with ACS were included. Baseline characteristics are summarized in Table 1. The median age ranged from 54.5 to 73 years, and 78.3% were male. Analysis of cardiovascular risk profile revealed that 22.7% had diabetes, 58.2% had dyslipidemia. 63.7% had hypertension, and 49% were ever smokers. STEMI was the predominant type of ACS, followed by NSTEMI and unstable angina. All patients were managed invasively. 1263 patients underwent OCT-guided and 1349 patients angiography-guided PCI. Stent implantation was the predominant revascularization strategy. The follow-up period ranged from 6 to 25 months.

### Primary outcome analysis

**Fig. 2:**
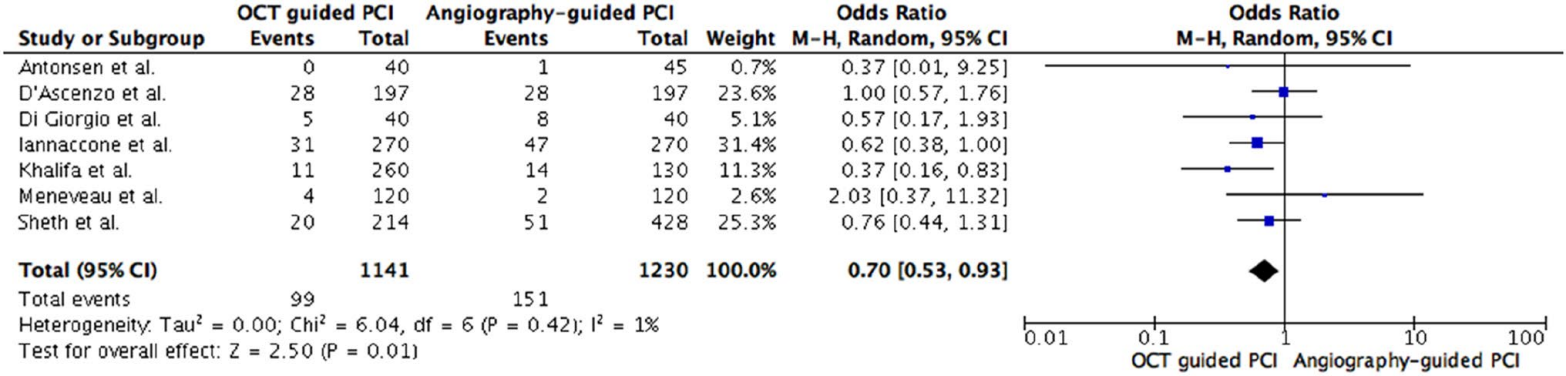
Primary analysis: Major adverse cardiac events. *OCT* optical coherence tomography, *PCI* percutaneous coronary intervention, *M-H* Mantel–Haenszel method, *CI* confidence interval

Seven trials reporting on 1141 patients treated with OCT-guided PCI compared to 1230 patients undergoing angiography-guided PCI were included in quantitative analysis of major adverse cardiac events [17–22, 24]. EROSION III did not explicitly report TVR and was excluded from primary outcome analysis [23]. MACE rate was 8.7% in OCT-guided PCI group compared to 12.3% in angiography-guided group. OCT guidance led to a significant difference with a 30% lower likelihood of MACE (OR 0.70, 95% CI 0.53–0.93, *p* = 0.01, *I*^2^ = 1%, non-relevant heterogeneity, see Fig. 2).

### Secondary outcome analyses

Overall, eight trials were included in secondary outcome analyses [17–24].

### All-cause mortality

**Fig. 3:**
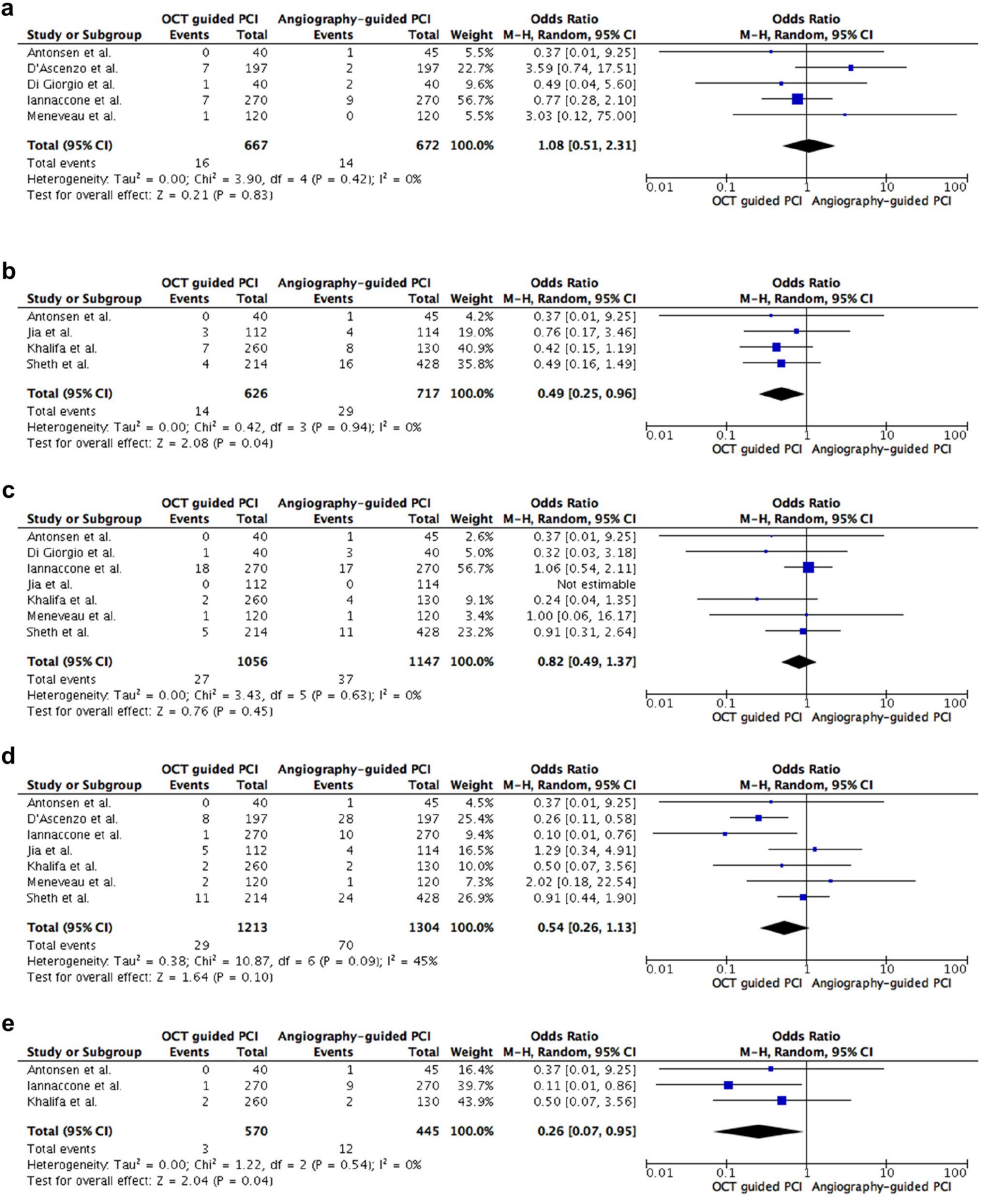
**a** Secondary analyses: all-cause mortality. **b** Secondary analyses: cardiac mortality. **c** Secondary analyses: myocardial infarction. **d** Secondary analyses: target vessel revascularization. **e** Secondary analyses: target lesion revascularization. *OCT* optical coherence tomography, *PCI* percutaneous coronary intervention, *M-H* Mantel–Haenszel method, *CI* confidence interval

Five trials reporting on 667 patients treated with OCT-guided PCI compared to 672 patients treated with angiography-guided PCI were analyzed [17, 18, 20, 21, 24]. All-cause mortality rate was 2.4% in OCT-guided PCI group compared to 2.1% in angiography-guided group without a statistical difference (OR 1.08, 95% CI 0.51–2.31, *p* = 0.83, *I*^2^ = 0%, non-relevant heterogeneity, see Fig. 3a).

### Cardiac mortality

Four trials reporting on 626 patients treated with OCT-guided PCI compared to 717 patients undergoing angiography-guided PCI were included [19, 22–24]. Cardiac mortality rate was 2.2% in OCT-guided PCI group compared to 4.0% in angiography-guided group. OCT-guided PCI led to a significant difference with a 51% lower likelihood of cardiac mortality (OR 0.49, 95% CI 0.25–0.96, *p* = 0.04, *I*^2^ = 0%, non-relevant heterogeneity, see Fig. 3b).

### Myocardial infarction

Seven trials reporting on 1056 patients treated with OCT-guided PCI compared to 1147 patients undergoing angiography-guided PCI were analyzed [17–19, 21–24]. Myocardial infarction rate was 2.6% in OCT-guided PCI group compared to 3.2% in angiography-guided group without a statistical difference (OR 0.82, 95% CI 0.49–1.37, *p* = 0.45, *I*^2^ = 0%, non-relevant heterogeneity, see Fig. 3c).

### Target vessel revascularization

Seven trials reporting on 1213 patients treated with OCT-guided PCI compared to 1304 patients undergoing angiography-guided PCI were included [18–24]. TVR rate was 2.4% in OCT-guided PCI group compared to 5.4% in angiography-guided group without a statistical difference (OR 0.54, 95% CI 0.26–1.13, *p* = 0.10, *I*^2^ = 45%, moderate heterogeneity, see Fig. 3d).

### Target lesion revascularization

Three trials reporting on 570 patients treated with OCT-guided PCI compared to 445 patients undergoing angiography-guided PCI were analyzed [21, 22, 24]. TLR rate was 0.5% in OCT-guided PCI group compared to 2.7% in angiography-guided group. OCT-guided PCI led to a significant difference with a 74% lower likelihood of TLR (OR 0.26, 95% CI 0.07–0.95, *p* = 0.04, *I*^2^ = 0%, non-relevant heterogeneity, see Fig. 3e).

Subgroup and sensitivity analyses did not demonstrate any statistically significant difference in primary or secondary outcomes (see Suppl. Table 1, Suppl. Table 2, and Suppl. Material 1).

## Discussion

To our knowledge, this is the first meta-analysis comprehensively assessing the effect of OCT-guided compared to angiography-guided PCI in patients with ACS. The main and novel findings of pooled data analyses were as follows:

- OCT-guided PCI resulted in a 30% lower likelihood of MACE and 74% lower odds of target lesion revascularization,
- OCT-guided PCI led to significant decrease of 51% in cardiac mortality, but this did not translate to difference in all-cause mortality.

Pooled data analysis is the uncontested strength of meta-analysis, but interpretation of overall effects requires cautious revision. On individual study level only Khalifa et al. demonstrated a significant MACE reduction and Iannaccone et al. exclusively reported a directed OR favoring OCT in TLR [21, 22]. All other trials did not show a significantly directed treatment effect according to MACE, cardiac death, or TLR on individual study level (see Figs. 2 and 3a–e). Moreover, in subgroup or sensitivity analyses the observed efficacy advantages were not thoroughly replicable. Study heterogeneity, study quality and low event rates might be possible explanations.

Then, pooled data analysis resulted in a significantly decreased cardiac mortality in OCT group, but all-cause mortality (OR 1.08, 95% CI 0.51–2.31) did not differ in overall analysis. Noteworthy, only OCTACS trial was eligible for both mortality analyses and this study itself lacked statistical power in mortality measurement [24]. Hence, one might speculate that structural interstudy heterogeneity rather than intervention itself might have contributed to the measured effects of all-cause or cardiac mortality. Important structural differences need to be acknowledged.

In included trials OCT was mostly used to examine deployed stents and assess success of revascularization [17–19, 23, 24]. One might hypothesize that the treatment benefit of OCT is mostly caused by its impact on optimizing revascularization strategy rather than its diagnostic role. In DOCTORS trial OCT imaging resulted in decision for stent optimization in 50% of patients and in 27% additional stents were implanted [18]. These data are in line with OCTACS and TOTAL each reporting 46% stent optimization rate following OCT [19, 24]. On the contrary, angiography led to further treatment in 22.5% of patients and additional stents were used in 18% [18]. However, the current study-level data set did not allow adequately powered subgroup calculation according to pre- or post-implantation OCT run (see suppl. Table 1). Consequently, this hypothesis cannot be validated. Potentially, a patient-level approach might add evidence whether the observed treatment effects of OCT are caused by stent optimization or whether competing mechanisms contribute to observed advantages.

Study design, performance, and study quality restrict generalizability to daily routine. Firstly, OCT was mainly performed by experienced operators and teams in designed trials. Secondly, OCT usage significantly increased procedural time (+ 13 min OCTACS, + 14 min EROSION, + 20 min TOTAL, + 20 min DOCTORS) and resulted in increased amount of contrast medium [18, 19, 23, 24]. Time delay might be harmful in urgent ACS scenario and higher contrast medium volume might decline kidney function [25]. The increased procedural duration and amount of contrast medium performing OCT was observed in elective setting likewise [13]. The EROSION III trial was intentionally designed to reduce stent implantation rate—86% of patients with plaque erosion were managed without stent implantation [23]. However, the majority of trials a priori used stenting as primary strategy to manage revascularization and subsequently used OCT not as diagnostic, but as implicit treatment tool.

The pooled meta-analysis pointed out weaknesses and gaps in the evidence. Upcoming controlled trials will not enroll all entities of ACS patients. They will not add substantial evidence to answer the current research question [26–28]. One might even speculate whether a RCT concomitantly enrolling STEMI and NSTEMI is reasonable given the fact that STEMI almost always requires immediate or rescue PCI [1].

Recently, implementation of the upcoming TACTICS registry was published [29]. This observational uncontrolled study will add further information on feasibility and diagnostic benefit of OCT-guided PCI in ACS patients, but underlies the limitations of a non-randomized study [29].

To definitely answer the research question future controlled studies are required. These trials should acknowledge the following terms to overcome the substantial bias domains:

- Comparable pretreatment
- Directed use of OCT, defined number of runs (if multiple runs are necessary), and limited operator’s discretion on treatment
- Precisely defined stenting conditions (Indications for stent deployment? How to treat complex lesions, calcified lesions or stenotic bifurcation? Does plaque erosion require stenting?)

In the absence of adequately powered, high-quality trials the use of OCT in ACS patients undergoing PCI revascularization cannot be generally recommended. Intracoronary imaging is useful, but implementation should not withhold restoration of coronary flow. An increase of procedural time and subsequent prolonged ischemia with loss of myocardium should not outweigh the potential benefits. Timing, the operator’s experience in imaging and interpretation, local resources, and patient’s characteristics like hemodynamic stability or renal function should be considered in decision making for OCT use in ACS.

### Limitations

Despite the methodological PRISMA approach, the current meta-analysis underlies inherent limitations. Specific aspects limiting generalizability should be acknowledged: we did not have access to patient-level data. Analysis of patient-level data would potentially produce more reasonable quantitative results, but was not applicable with limited access to only published data.

Patients were predominantly male and cardiovascular risk factors were unequally distributed. Two studies exclusively included STEMI patients, whereas two trials only enrolled NSTE-ACS patients. All studies were unblinded trials, and five were NRS. These facts might have contributed to selection, detection, and performance bias.

The follow-up duration and the timing of OCT runs varied between the trials. Three trials only investigated OCT use prior to stent implantation, whereas all other trials examined stent optimization strategy by OCT. Two trials did not report on stenting strategy and the EROSION III trial was conducted to reduce stent implantation rate by intracoronary imaging. These aspects indicate substantial performance bias.

The judgement of risk of bias on trial level demonstrated variance in study quality. This heterogeneity resulted in the authors’ decision to add further post hoc sensitivity analysis. The current meta-analysis only measured efficacy end points, safety outcomes like bleeding, or vascular access complication were not assessed.

## Conclusion

The evidence suggests that PCI guidance with OCT in ACS decreases MACE, cardiac death, and target lesion revascularization compared to angiography in pooled data analysis. On individual study level, in subgroup or sensitivity analyses these advantages were not thoroughly replicable. Study heterogeneity, study quality, and low event rates might be possible explanations.

Future randomized clinical trials with adequate statistical power and enrollment of all ACS entities are required to clarify the role of OCT as stent optimization in ACS revascularization procedures.

### Supplementary Information

Below is the link to the electronic supplementary material.

Supplementary file1 (DOCX 15 KB)

Supplementary file2 (DOCX 3967 KB)

Supplementary file3 (DOCX 28 KB)

## Abbreviations

ACS: Acute coronary syndrome
CI: Confidence interval
FFR: Fractional flow reserve
IVUS: Intravascular ultrasonography
MACE: Major adverse cardiac event
NRS: Non-randomized controlled studies
NSTE-ACS: Non-ST segment elevation acute coronary syndrome
NSTEMI: Non-ST segment elevation myocardial infarction
OCT: Optical coherence tomography
OFDI: Optical frequency domain imaging
OR: Odds ratio
PCI: Percutaneous coronary intervention
PSM: Propensity score matching
RCT: Randomized controlled trial
STEMI: ST segment elevation myocardial infarction
TLR: Target lesion revascularization
TVR: Target vessel revascularization
