# Supplementary material for: Optical coherence tomography-guided versus angiography-guided percutaneous coronary intervention in acute coronary syndrome: a meta-analysis
Source: Clin Res Cardiol. 2023 Jul 31;113(7):967–76. doi: 10.1007/s00392-023-02272-7 (PMC11219421; doi:10.1007/s00392-023-02272-7)
Supplement: Supplementary file 1 — Supplementary file1 (DOCX 15 KB) [file 392_2023_2272_MOESM1_ESM.docx]

**Suppl. Table 1: Subgroup analysis – OCT after stent implantation**

|  | **Studies included** | **Studies excluded** | **OCT guided PCI**  **Events/Patients** | **Angiography guided PCI**  **Events/Patients** | **Odds ratio (95% CI)** |
| --- | --- | --- | --- | --- | --- |
| **Major adverse cardiac events** | Antonsen et al (OCTACS)  Di Giorgio et al.  Meneveau et al. (DOCTORS)  Sheth et al. (TOTAL) | D’Ascenzo et al.  Iannaccone et al.  Khalifa et al. | 29/414 | 62/633 | 0.77 (0.48 to 1.24) |
| **All-cause mortality** | Antonsen et al (OCTACS)  Di Giorgio et al.  Meneveau et al. (DOCTORS) | D’Ascenzo et al.  Iannaccone et al. | 2/200 | 3/205 | 0.74 (0.14 to 3.90) |
| **Cardiac death** | Antonsen et al (OCTACS)  Jia et al. (EROSION III)  Sheth et al. (TOTAL) | Khalifa et al. | 7/366 | 21/587 | 0.55 (0.23 to 1.31) |
| **Myocardial infarction** | Antonsen et al (OCTACS)  Di Giorgio et al.  Jia et al. (EROSION III)  Meneveau et al. (DOCTORS)  Sheth et al. (TOTAL) | Iannaccone et al.  Khalifa et al. | 7/526 | 16/747 | 0.73 (0.30 to 1.77) |
| **Target vessel revascularization** | Antonsen et al (OCTACS)  Jia et al. (EROSION III)  Meneveau et al. (DOCTORS)  Sheth et al. (TOTAL) | D’Ascenzo et al.  Iannaccone et al.  Khalifa et al. | 18/486 | 30/707 | 1.00 (0.54 to 1.84) |
| **Target lesion revascularization** | Antonsen et al (OCTACS) | Iannaccone et al.  Khalifa et al. | 0/40 | 1/45 | Not applicable |
| Legend:  OCT: optical coherence tomography; PCI: percutaneous coronary intervention | | | | | |

**Suppl. Table 2: Sensitivity analyses according to risk of bias**

|  | **Studies included** | **Studies excluded** | **OCT guided PCI**  **Events/Patients** | **Angiography guided PCI**  **Events/Patients** | **Odds ratio (95% CI)** |
| --- | --- | --- | --- | --- | --- |
| **Major adverse cardiac events** | D’Ascenzo et al.  Iannaccone et al.  Meneveau et al. (DOCTORS)  Sheth et al. (TOTAL) | Antonsen et al (OCTACS)  Di Giorgio et al.  Khalifa et al. | 83/801 | 128/1015 | 0.78 (0.58 to 1.06) |
| **All-cause mortality** | D’Ascenzo et al.  Iannaccone et al.  Meneveau et al. (DOCTORS) | Antonsen et al (OCTACS)  Di Giorgio et al. | 15/587 | 11/587 | 1.50 (0.49 to 4.62) |
| **Cardiac death** | Jia et al. (EROSION III)  Sheth et al. (TOTAL) | Antonsen et al (OCTACS)  Khalifa et al. | 7/326 | 20/542 | 0.57 (0.23 to 1.40) |
| **Myocardial infarction** | Iannaccone et al.  Jia et al. (EROSION III)  Meneveau et al. (DOCTORS)  Sheth et al. (TOTAL) | Antonsen et al (OCTACS)  Di Giorgio et al.  Khalifa et al. | 24/716 | 29/932 | 1.01 (0.58 to 1.79) |
| **Target vessel revascularization** | D’Ascenzo et al.  Iannaccone et al.  Jia et al. (EROSION III)  Meneveau et al. (DOCTORS)  Sheth et al. (TOTAL) | Antonsen et al (OCTACS)  Khalifa et al. | 27/913 | 67/1129 | 0.56 (0.22 to 1.39) |
| **Target lesion revascularization** | Iannaccone et al. | Antonsen et al (OCTACS)  Khalifa et al. | 1/270 | 9/270 | Not applicable |
| Legend:  OCT: optical coherence tomography; PCI: percutaneous coronary intervention | | | | | |
