# Supplementary material for: Optical coherence tomography-guided versus angiography-guided percutaneous coronary intervention in acute coronary syndrome: a meta-analysis
Source: Clin Res Cardiol. 2023 Jul 31;113(7):967–76. doi: 10.1007/s00392-023-02272-7 (PMC11219421; doi:10.1007/s00392-023-02272-7)
Supplement: Supplementary file 2 — Supplementary file2 (DOCX 3967 KB) [file 392_2023_2272_MOESM2_ESM.docx]

**Suppl. Figure 1a: Major adverse cardiac events – OCT after stent implantation**


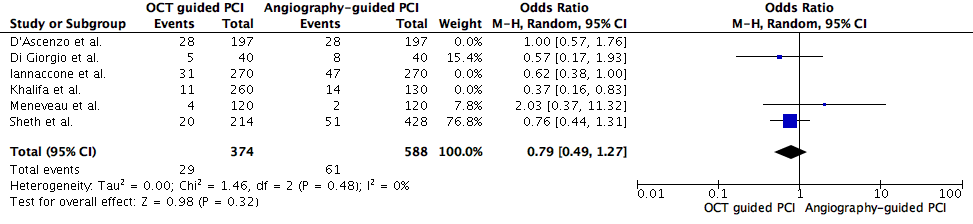


**Suppl. Figure 1b: All cause mortality – OCT after stent implantation**

**
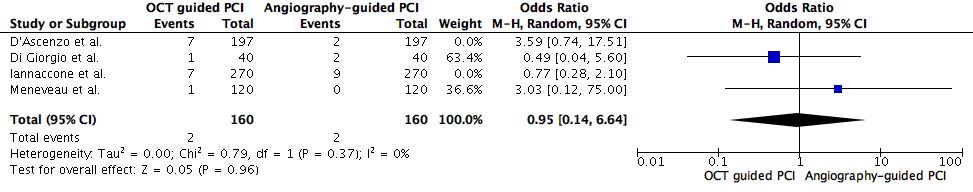
**

**Suppl. Figure 1c: Cardiac mortality – OCT after stent implantation**

**
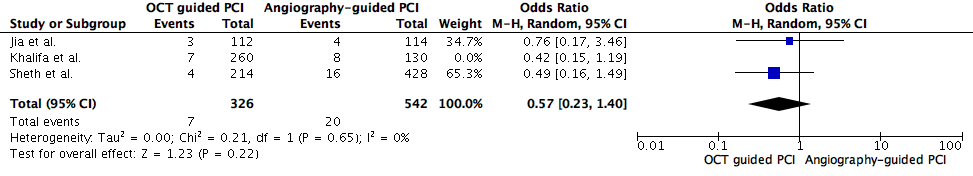
**

**Suppl. Figure 1d: Myocardial infarction – OCT after stent implantation**

**
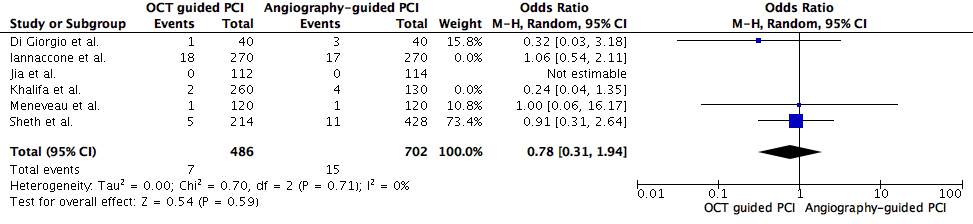
**

**Suppl. Figure 1e: Target vessel revascularization – OCT after stent implantation**

**
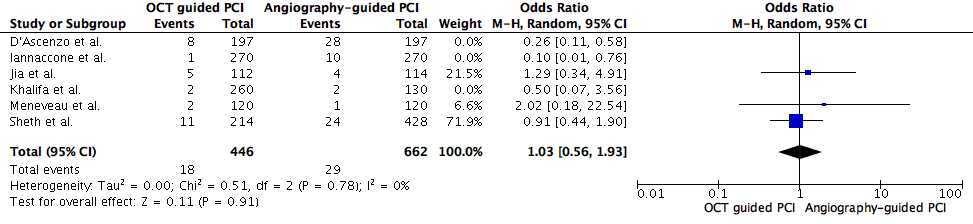
**
