# Supplementary material for: Optical coherence tomography-guided versus angiography-guided percutaneous coronary intervention in acute coronary syndrome: a meta-analysis
Source: Clin Res Cardiol. 2023 Jul 31;113(7):967–76. doi: 10.1007/s00392-023-02272-7 (PMC11219421; doi:10.1007/s00392-023-02272-7)
Supplement: Supplementary file 3 — Supplementary file3 (DOCX 28 KB) [file 392_2023_2272_MOESM3_ESM.docx]

**Suppl. material 1:**

**Detailed description of methodological approach**

*Search description and study selection*

The systematic literature search was performed in three data bases including Medline (via PubMed), Web of Science and Cochrane Library. The search syntax consisted of the following terms: “percutaneous coronary intervention”, “PCI”, “intravascular imaging”, “optical coherence tomography”, “OCT”, “optical frequency domain imaging”, “OFDI”, “acute coronary syndrome” and “ACS”. These search terms were connected with Boolean operators.

The first search was performed on October, 31th 2022. The last search update was carried out on January, 12th 2023. No restrictions on publication date, language or study size were applied. After exclusion of duplicates and screening of titles and abstracts according to the eligibility criteria, full texts of the remaining articles were assessed.

The study selection was independently performed by two reviewers (SM, MMM). In case of any disagreement, this was resolved by consensus with the senior author (SL).

All entities of ACS were included (STEMI, NSTEMI, unstable angina pectoris). No restrictions were applied for follow-up duration. Double publications, case reports, case series without control groups, reviews and conference abstracts were excluded. Studies with solely conservative treatment like guideline directed medical therapy (GDMT) as comparator to OCT guided PCI were not eligible for analysis.

*Data extraction*

Data were extracted by one investigator (SMM) using a standardized pre-specified data collection form. Main study reports as well as any supplementary appendices were reviewed. Pre-specified data elements included study design, patient baseline characteristics, procedural and follow-up data.

**Results of subgroup and sensitivity analyses**

*Subgroup analysis*

We grouped the included trials according to the OCT run strategy in post-hoc subgroup analysis. Patients undergoing OCT after stent implantation with intention of stent optimization were defined as subsequent subgroup of interest (17-19,23,24). Patients with OCT as diagnostic approach only were excluded. Pooled data on TLR was not applicable because only one trial could be included (24). Subgroup analyses did not demonstrate any statistically significant difference in primary or secondary outcomes (see suppl. table 1).

*Sensitivity analyses*

We added post-hoc sensitivity analyses investigating the potential impact of study quality on outcome investigation. Two NRS were excluded due to critical risk of bias (17,22). The randomized OCTACS trial was judged to have high risk of bias and was excluded, too (24). There was no statistically significant difference in primary or secondary outcomes (see suppl. table 2). Pooled data on TLR was not applicable because only one trial could be included (21).
